# Quantitative proteomics for identifying biomarkers for tuberculous meningitis

**DOI:** 10.1186/1559-0275-9-12

**Published:** 2012-11-30

**Authors:** Ghantasala S Sameer Kumar, Abhilash K Venugopal, Anita Mahadevan, Santosh Renuse, H C Harsha, Nandini A Sahasrabuddhe, Harsh Pawar, Rakesh Sharma, Praveen Kumar, Sudha Rajagopalan, Keith Waddell, Yarappa L Ramachandra, Parthasarathy Satishchandra, Raghothama Chaerkady, T S Keshava Prasad, K Shankar, Akhilesh Pandey

**Affiliations:** 1 Institute of Bioinformatics, International Technology Park, Bangalore, 560066, India; 2 Department of Biotechnology, Kuvempu University, Shimoga, 577451, India; 3 McKusick-Nathans Institute of Genetic Medicine, Johns Hopkins University, 733 N. Broadway, BRB 527, Baltimore, MD, 21205, USA; 4 Departments of Biological Chemistry, Johns Hopkins University School of Medicine, Baltimore, MD, 21205, USA; 5 Department of Neuropathology, National Institute of Mental Health and Neurosciences, Bangalore, 560029, India; 6 Amrita School of Biotechnology, Amrita Vishwa Vidyapeetham, Kollam, 690525, India; 7 Manipal University, Madhav Nagar, Manipal, 576104, India; 8 Rajiv Gandhi University of Health Sciences, Bangalore, 560041, India; 9 Department of Neurochemistry, National Institute of Mental Health and Neurosciences, Bangalore, 560029, India; 10 Agilent Technologies India Pvt. Ltd, Bangalore, 560048, India; 11 Agilent Technologies, Santa Clara, CA, USA; 12 Department of Neurology, National Institute of Mental Health and Neurosciences, Bangalore, 560029, India; 13 Centre of Excellence in Bioinformatics, School of Life Sciences, Pondicherry University, Pondicherry, 605014, India; 14 Pathology Johns Hopkins University School of Medicine, Baltimore, MD, 21205, USA; 15 Oncology, Johns Hopkins University School of Medicine, Baltimore, MD, 21205, USA

**Keywords:** Relative quantitation, Cerebrospinal fluid, Histopathology, Early diagnosis, Tuberculosis

## Abstract

**Introduction:**

Tuberculous meningitis is a frequent extrapulmonary disease caused by *Mycobacterium tuberculosis* and is associated with high mortality rates and severe neurological sequelae. In an earlier study employing DNA microarrays, we had identified genes that were differentially expressed at the transcript level in human brain tissue from cases of tuberculous meningitis. In the current study, we used a quantitative proteomics approach to discover protein biomarkers for tuberculous meningitis.

**Methods:**

To compare brain tissues from confirmed cased of tuberculous meningitis with uninfected brain tissue, we carried out quantitative protein expression profiling using iTRAQ labeling and LC-MS/MS analysis of SCX fractionated peptides on Agilent’s accurate mass QTOF mass spectrometer.

**Results and conclusions:**

Through this approach, we identified both known and novel differentially regulated molecules. Those described previously included signal-regulatory protein alpha (SIRPA) and protein disulfide isomerase family A, member 6 (PDIA6), which have been shown to be overexpressed at the mRNA level in tuberculous meningitis. The novel overexpressed proteins identified in our study included amphiphysin (AMPH) and neurofascin (NFASC) while ferritin light chain (FTL) was found to be downregulated in TBM. We validated amphiphysin, neurofascin and ferritin light chain using immunohistochemistry which confirmed their differential expression in tuberculous meningitis. Overall, our data provides insights into the host response in tuberculous meningitis at the molecular level in addition to providing candidate diagnostic biomarkers for tuberculous meningitis.

## Introduction

Tuberculosis (TB) is a common and sometimes fatal transmissible disease, especially in developing countries. Approximately thirty percent of the global population is exposed to the acid-fast bacilli causing TB. Of those who are infected with tuberculosis, ~10% percent develop a clinical manifestation of the disease during their lifetime. From a global perspective, approximately twenty percent of TB infected population live in India. The World Health Organization (*WHO*) has estimated that one million children develop TB annually worldwide which accounts for about 11% of all TB cases [1]. Tuberculous bacilli most commonly infect lungs. *Mycobacterium tuberculosis* (MTB) may also spread to extrapulmonary sites including the meninges, lymph nodes, genitourinary tract, skeletal system and skin [2]. Tuberculous meningitis (TBM) is the infection of meninges caused by MTB, with a mortality rate of ~30%. Further, those who survive TBM are usually left with severe neurological defects [3-5]. There is an increased risk of TBM in HIV-infected patients as compared to non-HIV infected cases although the clinical manifestations of the disease do not differ between the two groups [6,7].

Culturing mycobacteria and subsequent microbiological examination is considered a gold standard for the diagnosis of TBM. However, this method is time consuming and insensitive, with a positive outcome achieved only in 25–70% of clinically diagnosed cases [8]. Although PCR assays can be an alternative rapid approach for diagnosis, they cannot differentiate between latent or active forms of the disease. Although nucleic acid amplification test (NAAT) has a high specificity when tested in body fluids, it lacks adequate sensitivity in cases of meningitis and pleuritis [9].

Amongst the existing molecular markers, Adenosine deaminase isoenzyme-2 (ADA2) has a sensitivity of 100% and a specificity of 86.4% for the detection of TBM in cerebrospinal fluid (CSF) [10]. Adenosine deaminase (ADA) activity in the CSF of TBM patients has been suggested to be useful for early differential diagnosis of TBM [11]. The ADA activity of CSF and plasma have been evaluated as a diagnostic aid in TBM [12] and ADA activity in CSF was considered to be a simple, useful and rapid diagnostic test for early recognition of TBM in children [13]. However, overexpression of ADA was also often overexpressed in other forms of meningitis including infections with pyogenic bacteria [14]. In the CSF of TBM patients, the presence of 65 kDa heat shock protein antigen might be a marker for early diagnosis of the disease [15]. High levels of CSF lactate and lactate dehydrogenase levels have also been suggested for diagnosing TBM [16].

Early diagnosis of TBM is considered a key to effective treatment and prognosis. Approximately 90% of the patients are diagnosed in stage II or III [17]. Overall, the diagnosis of TBM still remains a major challenge due to inadequate current diagnostic methods and poor sensitivity and/or specificity of existing markers. Although corticosteroids are used extensively to reduce mortality and neurological disability, it may not be the only solution to reduce the mortality and morbidity [18]. In TBM, a number of pathological changes including meningeal adhesion, infarction, tuberculoma and hydrocephalus may occur leading to neurological sequelae [4]. These sequelae are known to correlate with the stage of meningitis at admission. Patients treated at an early stage have a five times higher chance of recovery than those with advanced disease stages [19]. Therefore, patient’s clinical condition at admission and delay in starting the treatment are important factors for determining their survival [20]. These findings emphasize the need to focus on identifying candidate molecular markers which can be developed as diagnostic tools in the management of TBM.

Mass spectrometry-based quantitative proteomics has emerged as a powerful approach for identifying and studying disease biomarkers and has become one of the essential tools in biomarker discovery [21,22]. Advances in quantitative mass spectrometry have led to identification and quantitation of biomarkers which serve as indicators of disease progression, prognosis, drug safety and help to elucidate the mechanism of drug treatment [23]. There are various labeling approaches that one can employ to carry out quantitative proteomic measurements. *In vitro* labeling methods include Isobaric Tags for Relative and Absolute Quantitation (iTRAQ), Isotope-Coded Affinity Tags (ICAT), ^**18**^O labeling and *in vivo* methods include Stable Isotope Labeling by Amino acids in Cell culture (SILAC) and ^**15**^Nlabeling [24,25]. iTRAQ labeling is an effective method for studying differential protein expression levels in tissue samples. It has been extensively used for biomarker discovery in various disease contexts [26-34].

In this study, we used an iTRAQ-based quantitative proteomic approach to identify differentially expressed proteins from brain tissues of tuberculous meningitis cases as compared to controls. We identified several proteins which are differentially expressed in TBM. These proteins include both novel and previously reported candidate protein markers. We validated some of these candidate biomarkers using immunohistochemical labeling.

## Materials and methods

### Sample collection

The study was approved by scientific ethics committee of National Institute of Mental Health and Neuro Sciences (NIMHANS), Bangalore, India. Samples from frontal cortex with overlying meninges from cases of tuberculous meningitis (n=6) and similar, but uninfected, control brain tissues (n=6) from victims of road traffic accidents were collected at the time of autopsy. The autopsy was conducted within 8–16 h postmortem with the body kept at 4°C after death. The samples were obtained from the Human Brain Tissue Repository, Department of Neuropathology, NIMHANS, Bangalore. Sample details including the selection criteria are provided in Additional file 1: Table S1.

### Sample preparation and iTRAQ labeling

Brain tissue samples were lysed in 0.5% SDS, sonicated, homogenized and centrifuged at 13,000 rpm for 10 min at 4°C. Supernatant was collected and protein quantitation was carried out by Lowry’s assay (Bio-Rad Hercules, CA; USA). For each condition, 160 μg of protein sample was utilized for the experiment. Each sample was treated with 4 μL of reducing agent (tris (2-carboxyethyl) phosphine (TCEP)) at 60°C for 1 h and alkylated with 2 μL of cysteine blocking reagent, methyl methanethiosulfonate (MMTS) for 10 min at room temperature. After alkylation, the samples were subjected to trypsin digestion (Sequencing Grade Modified Trypsin, Promega Cat#:V511A) using 1:20 (w/w) at 37°C for 16 h. Peptide samples from each condition was split in equal halves (80 μg each) and labeled with iTRAQ 4-plex reagents (catalog # 4352135, Applied Biosystems, Foster City, CA, USA) as per manufacturer’s protocol. We used technical replicates for control and TBM samples. Peptides from control samples were labeled with iTRAQ reagents yielding reporter ions 114 and 115 while peptides from TBM were labeled with 116 and 117. The samples were pooled following iTRAQ labeling.

### Strong cation exchange chromatography (SCX)

Pooled iTRAQ labeled peptides were fractionated by strong cation exchange chromatography on PolySULFOETHYL A column (200 x 2.1 mm; 5 μm; 200Å PolyLC, Columbia, MD) using Agilent’s 1200 series HPLC system. The peptides were reconstituted in SCX solvent A (10 mM potassium phosphate, 25% acetonitrile, pH 2.8) and loaded on SCX column isocratically using 100% solvent A for 20 min at a flow rate of 200 ul per minute. Peptides were eluted using a 30 min gradient from 8% to 35% solvent B (350 mM KCl in solvent A). Fractions were collected every minute using a fraction collector. The fractions were vacuum dried and stored at −80°C until LC-MS/MS analysis.

### LC-MS/MS analysis

The samples were analyzed on HPLC chip-cube interfaced with Accurate Mass 6520 quadrupole time of flight mass (QTOF) spectrometer (Agilent Technologies, Santa Clara, CA). The HPLC-Chip contains a 40 nl enrichment column and 43 mm x 75 um analytical column. These columns were made up of a reversed-phase material Zorbax 300SB-C_18_, with a particle size of 5 μm. The samples were loaded onto the enrichment column using Agilent’s 1200 series capillary liquid chromatography pump at a flow rate of 3 μl/min using 97% solvent A and 3% solvent B. An injection flush volume of 4 μl was applied during enrichment step. The peptides were eluted at a flow rate of 400 nl/min using a gradient of solvent A (0.1% formic acid) and solvent B (0.1% formic acid in 90% acetonitrile). The gradient was started from 3% to18% of solvent B over 8 min, subsequently changed to 22% B in the next 7 min and finally changed to 45% of solvent B for 25 min. MassHunter workstation data acquisition software (Version B.01.03) was used for data dependent acquisition. MS spectra were acquired for 1 second from m/z 350–1800 followed by three MS/MS spectra in next second comprising the duty cycle of 2.1 second including an interscan delay of 0.1 second. Precursor ions were preferred based on charge state in the order of 2+, 3+ and >3+. The capillary and fragmentor voltage of 1950V and 175 V respectively was applied with a medium isolation width of 4 m/z and a collision energy slope of 3 V plus at offset of 2 V.

### Data analysis

The mass spectrometry raw data was processed to peak list format by using MassHunter Qualitative Analysis software (Agilent Technologies, Version B.03.3). These processed peak list files were then searched against Human RefSeq Protein Database (Release 40) containing 31,811 protein sequences through Proteome Discoverer platform (Thermo Scientific, Version 1.2). The workflow includes spectrum files, spectrum selector, Mascot and Sequest search nodes followed by peptide validator for false discovery analysis whereas a reporter ion quantifier was used for quantitation. Search parameters included trypsin as the enzyme with one missed cleavage allowed, oxidation of methionine, deamidation at aspargine and glutamine were set as a variable modification whereas methyl-thio at cysteine and iTRAQ label at N-terminus of the peptide and lysine were set as a fixed modification. The reporter ion window tolerance was set at 100 ppm. The identified peptides were filtered using 1% false discovery rate derived using decoy database search strategy and top ranked hit based on peptide score, XCorr and IonScore for Sequest and Mascot respectively. The search results from both Masoct and Sequest were merged and unique peptide(s) identified for each protein were used to calculate relative protein quantitation in Proteome Discoverer workflow. The average ratio was used for relative protein quantification for proteins with multiple peptide matches. Bioinformatic analysis was carried out to categorize proteins based on biological processes, cellular component and molecular function classification using annotations in Human Protein Reference Database (HPRD, http://hprd.org) [35], which is in compliance with gene ontology (GO) standards.

### Immunohistochemical labeling

Formalin fixed and paraffin embedded autopsy tissues were collected and cut into 4 μm thick sections on glass slides. These slides were subjected for deparaffinization and rehydration. Endogenous peroxidase activity was quenched by 3% H_2_O_2_ for 20 min at room temperature. For antigen retrieval, the tissue sections were microwaved in citrate buffer (pH 6.0) for 30 min. The tissue sections were incubated with 3% skimmed milk in PBS, pH 7.4 at room temperature. The tissue sections were incubated with primary antibodies at following dilutions - anti-amphiphysin (dilution 1:100, catalog # ab52646), anti-neurofascin (dilution1:250, catalog # ab31457), anti-ferritin light chain (dilution 1:500, catalog # ab69090) which were purchased from Abcam (Cambridge UK). In parallel with test slides, negative and positive controls were also subjected for overnight incubation at room temperature, followed by incubation with prediluted secondary antibody conjugated with poly HRP (catalog # K4011) from Dako. The reaction was visualized with chromogen substrate DAB/H2O2 (Dako catalog # K4007) as per manufacturer’s instructions. The sections were counterstained with hematoxylin.

## Results and discussion

**Figure 1:**
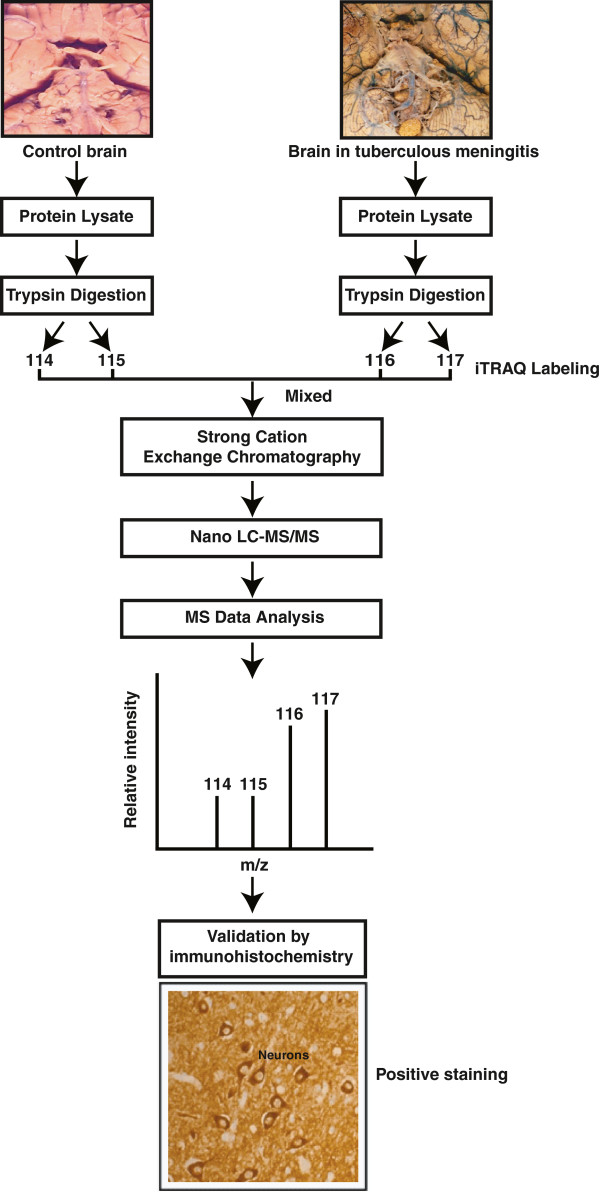
**Outline of the quantitative proteomic strategy.** TBM-infected and uninfected frontal cortex tissues were used to extract proteins and subjected to digestion with trypsin. Peptides from the two groups were labeled with iTRAQ reagents followed by LC-MS/MS on a QTOF mass spectrometer. A subset of the differentially expressed molecules were validated by immunohistochemistry.

**Figure 2:**
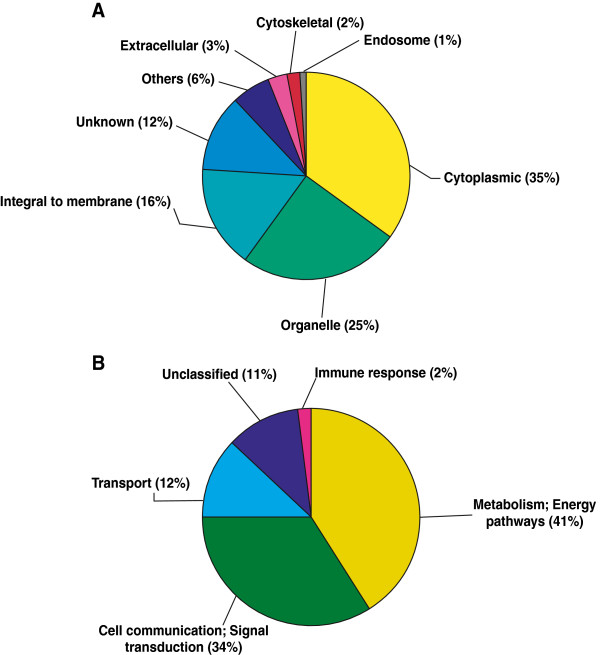
**Subcellular localization and biological process of differentially expressed proteins.** Subcellular localization (**A**) and biological process (**B**) of differential expressed proteins are shown.

We used six TBM and six uninfected samples for our quantitative proteomic analysis. The overall workflow for the study is depicted in Figure 1. The tandem mass spectra were searched against human NCBI RefSeq release 40 (31,811 protein sequences) using Mascot and Sequest search engines through the Proteome Discoverer software. A total of 81,494 spectra were acquired which resulted in 5,988 peptide-spectrum matches. We identified a total of 434 proteins with 919 unique peptides. The protein and peptide data is provided in Additional file 2: Table S2 and Additional file 3: Table S3, respectively. In all, we identified 110 upregulated and 24 downregulated proteins at 1% FDR. The distribution of cellular localization and biological processes of the differentially expressed proteins is shown in Figure 2A and B. The subcellular localization and biological processes of the differentially expressed proteins were classified based on annotations in Human Protein Reference Database (HPRD, http://hprd.org) [35]. Partial list of differentially expressed proteins are provided in Tables 1 and 2.

**Table 1:** A partial list of proteins overexpressed in TBM

	**Gene Symbol**	**Protein**	**Description**	**Fold-change**
1	*WARS*	Tryptophanyl-tRNA synthetase, cytoplasmic isoform b	It induced by interferon and involved in the catalyzation of the aminoacylation of tRNA (trp) with tryptophan.	7.2
2	*SLC4A4*	Electrogenic sodium bicarbonate cotransporter 1 isoform 2	It plays a functional role in the regulation of bicarbonate secretion and absorption and intracellular pH.	6.9
3	*NPM1*	Nucleophosmin isoform 3	It is a phosphoprotein which shuttles between the nucleus and the cytoplasm. It plays a role in ARF/p53 signaling pathway.	4.8
4	*DHX9*	ATP-dependent RNA helicase A	It localizes to cytoplasm and nucleus. It acts as a transcriptional regulator.	4.9
5	*HPCA*	Neuron-specific calcium-binding protein hippocalcin	It belongs to the neuron-specific calcium-binding proteins family. It may play a role in the neurons of the central nervous system.	4.4
6	*GPM6A*	Neuronal membrane glycoprotein M6-a isoform 3	It is a transmembrane protein and expressed on neurons in the central nervous system.	3.8
7	*GFAP*	Glial fibrillary acidic protein isoform 2	It is an intermediate filament protein and a marker for astrocytes. It is overexpressed in astrogliosis.	3.2
8	*SLC8A2*	Sodium/calcium exchanger 2 precursor	It is a Sodium/calcium exchanger, regulates the intracellular calcium concentrations.	2.8
9	*VIM*	Vimentin	It belongs to the intermediate filament protein family. It plays a role in cell shape and integrity.	2.8
10	*VGF*	VGF nerve growth factor inducible precursor	It expressed in neuroendocrine cells and overexpressed by nerve growth factor	2.5

**Table 2:** A partial list of downregulated proteins in TBM

	**Gene Symbol**	**Protein**	**Description**	**Fold-change**
1	*PDIA6*	Protein disulfide-isomerase A6 precursor	It is endoplasmic reticulum (ER) resident protein and it plays a role in folding of disulfide-bonded proteins	2.0
2	*KPNA4*	Importin subunit alpha-4	It is cytoplasmic protein, recognizes nuclear localization signals.	2.0
3	*PFDN5*	Prefoldin subunit 5 isoform alpha	It belongs to the prefoldin alpha subunit family. It is a subunit of the molecular chaperone complex, involved in protein folding.	2.5
4	*GARS*	Glycyl-tRNA synthetase	It belongs to the class II family of tRNA synthetases and this protein plays a role in autoimmune diseases.	3.0
5	*MDH1*	Malate dehydrogenase, cytoplasmic	It localized to the cytoplasm and mitochondria. It involved in malate-aspartate shuttle.	3.0
6	*AK1*	Adenylate kinase isoenzyme 1	It is an enzyme involved in regulating the adenine nucleotide composition in the cell and it is localized in the cytosol.	3.0
7	*LANCL1*	LanC-like protein 1	It is a loosely associated peripheral membrane protein which belongs to the LanC family of bacterial membrane-associated proteins. It plays a role in antimicrobial peptide synthesis.	5.0
8	*SNCG*	Gamma-synuclein	It belongs to the member of the synuclein family of proteins and it may play a role in the pathogenesis of neurodegenerative diseases.	5.0
9	*PTPLB*	Protein-tyrosine phosphatase-like member B	It localizes to endoplasmic reticulum (ER) and involved in the dehydration of very long chain fatty acid synthesis	7.0
10	*NCDN*	Neurochondrin isoform 2	It is a cytoplasmic protein and may play a role in spatial learning processes.	7.0

### Proteins previously implicated in TBM and tuberculosis associated studies

**Figure 3:**
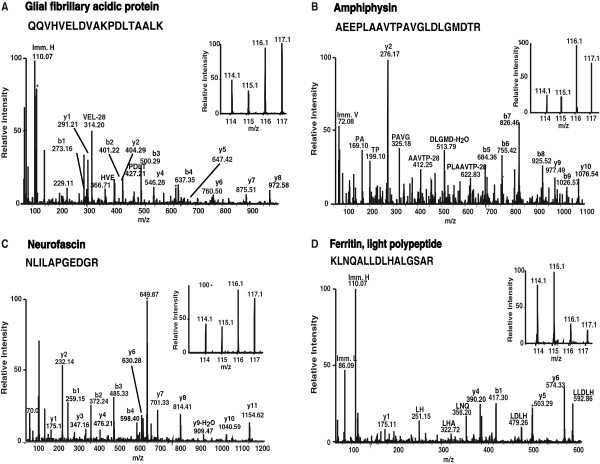
**Representative MS and MS/MS spectra of upregulated as well as downregulated proteins.** MS and MS/MS spectra of peptides from selected differentially expressed proteins identified in this study. Insets shows relative intensities of reporter ions. (**A**) Glial fibrilllary acid protein (GFAP); (**B**) Amphiphysin (AMPH); (**C**) Neurofascin (NFASC) (**D**) Ferritin, light chain (FTL).

Proteins that have previously been implicated in TBM and tuberculosis associated studies included Vimentin (VIM), Peroxiredoxin 5 (PRDX5*),* Glial fibrillary acidic protein (GFAP), Signal-regulatory protein alpha (SIRPA) and Protein disulfide isomerase family A, member 6 (PDIA6). VIM is an intermediate filament family protein and is also the potential ligand for natural cytotoxicity triggering receptor 1 (NKp46)*.* VIM was shown to be 2.8-fold upregulated in the present study. VIM is involved in the lysis of the MTB-infected cells through NKp46 [36,37]. PRDX5 is a member of the peroxiredoxin family of antioxidant enzymes. *PRDX5* was found to be overexpressed in MTB (H37Rv) infected mice study [38]. In our study, it was found to be 2.2-fold overexpressed in TBM as compared to controls. PRDX5 plays an antioxidant protective role in tissues. GFAP showed 3.2-fold upregulation in TBM as compared to controls. Our finding correlated with a recent study that described a 4.7-fold overexpression of *GFAP* at mRNA level in TBM cases [39]. Representative MS/MS and reporter ion spectra of differentially regulated molecules are provided in Figure 3. MS/MS spectrum of GFAP is depicted in Figure 3A. SIRPA belongs to the signal regulatory family. The regulation of SIRPA expression can change the effectiveness of signaling processes. Growth factor receptors and growth hormone receptor signaling is suppressed by the upregulation of *SIRPA*[40-43]. SIRPA regulates the NF*k*B activity that renders the cells resistant to TNF mediated apoptosis [44]. A recent study reported *SIRPA* to be 2-fold upregulated at the mRNA level in TBM [39]. In our study, also SIRPA protein was found to be 2-fold overexpressed in TBM. Protein disulfide isomerase family A, member 6 (PDIA6) is a protein that belongs to the endoplasmic reticulum (ER) and it is involved in the folding of disulphide bonded proteins [45]. This protein was shown to be downregulated (2.6-fold) in TBM at the mRNA level [39] and confirmed to be downregulated (2.5-fold) at the protein level in our study.

In our previous study on TBM using gene expression microarray, we had identified 2,434 differentially regulated transcripts [39]. In current proteomic study, 33 out of 134 differentially regulated proteins were also identified to be differentially expressed in the microarray dataset. However, only three proteins (GFAP, SIRPA, ACTB) were found to be correlated at both mRNA and protein levels, which is likely due to low correlation frequently observed between transcriptomic and proteomic studies.

### Differentially expressed proteins with no previous association with TBM

We identified a large number of differentially expressed proteins, which have not been reported earlier in the literature to be associated with TBM. These novel proteins include Ca++−dependent secretion activator 2 (CADPS2) which belongs to the calcium-dependent activator of secretion (CAPS) protein family, and was found 3-fold upregulated in the present study. CADPS2 protein regulates the exocytosis of synaptic and dense-core vesicles in neurons and neuroendocrine cells [46,47]. Amphiphysin (AMPH) (Figure 3B) is an adapter molecule which plays a role in synaptic vesicle endocytosis [48] and it was found to be 3.7-fold overexpressed in TBM compared to control cases. Heat shock protein 90kDa alpha (cytosolic), class A member 1 (HSP90AA1) is a highly conserved molecular chaperone and was found to be 2.1-fold upregulated in the present study. HSP90AA1 is a molecular chaperone, which plays a role in signal transduction, protein degradation, protein folding and is expressed under stress conditions and according to a recent report it plays a role in gene expression in mammalian cells [49]. Neurofascin (NFASC) (Figure 3C) is the L1 family immunoglobulin cell adhesion molecule, found to be 2-fold upregulated in the current study. NFASC plays a role in organization of the axon initial segment (AIS) and nodes of Ranvier in central nervous system, neurite extension and neurite fasciculation [50-52]. Ferritin, light chain (FTL) (Figure 3D) protein is an iron storage protein which, play a role in neurodegeneration [53] and found to be 66-fold downregulated in the present study. Downregulation of FTL causes the depigmentation in metastatic melanoma cells [54] and responds during inflammation especially where oxidative stress and reactive oxygen intermediates are generated [55,56].

### Validation of candidate biomarkers by immunohistochemical labeling

We used immunohistochemistry (IHC) to validate a subset of differentially expressed proteins identified in iTRAQ-based study. The results from IHC validation of AMPH, NFASC and FTL from fifteen TBM cases are summarized below.

**Figure 4:**
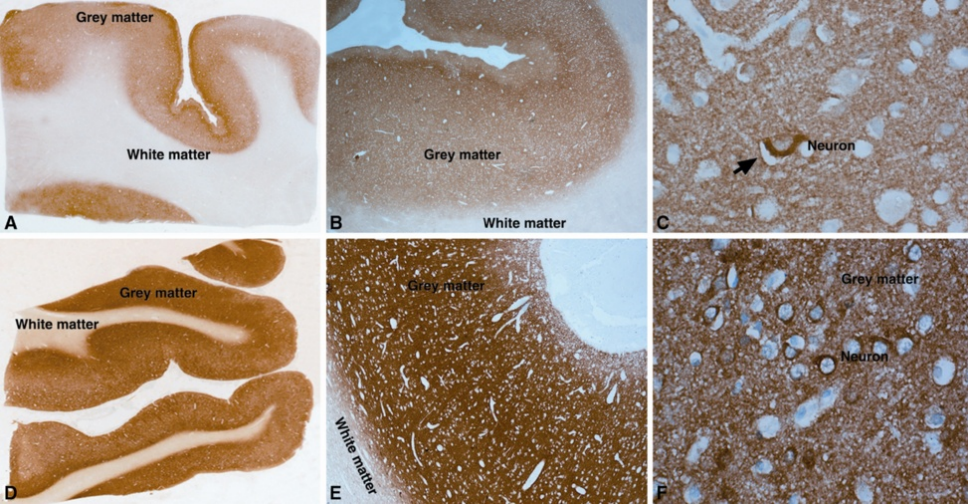
**Immunohistochemical labeling of AMPH protein overexpressed in TBM.** Normal controls (**A**-**C**): Whole mount preparation of cerebral cortex (**A**) shows labeling of cortical ribbon in a synaptic pattern (**B**). Higher magnification highlights scattered strong labeling of pyramidal neurons (arrow, **C**) with synaptic pattern of neuropil labeling. TBM (**D**-**F**): Strong labeling of the cortical ribbon seen in contrast to controls (**D**) with intense labeling of neuropil in grey matter (**E**) and pyramidal neurons in the cortex (**F**). [**A**, **D**: whole mounts x10, **B**, **E**: Obj x5, **C**: Obj x40, **F**: Obj x 20] [g – grey matter, w – white matter].

Amphiphysin (AMPH) is an adapter molecule associated with cytoplasmic surface of the synaptic vesicle, which was confirmed to be overexpressed in all 15 TBM cases tested (Figure 4). AMPH protein expression was seen diffusely distributed in synaptic fashion in the cortical ribbon in control cases (Figure 4A, B). The neurons in the grey matter showed cytoplasmic labeling with AMPH (Figure 4C). In comparison, cases of tuberculous meningitis revealed intense staining of the neuropil of the cortical ribbon reflecting upregulation of protein expression (Figure 4D, E). Several neurons showed cytoplasmic accumulation of protein within the neuronal soma (Figure 4F). Astrocytes and microglia were not labeled and white matter was devoid of labeling. Inflammatory exudates in the meninges in the cases of tuberculous meningitis also did not reveal positivity.

**Figure 5:**
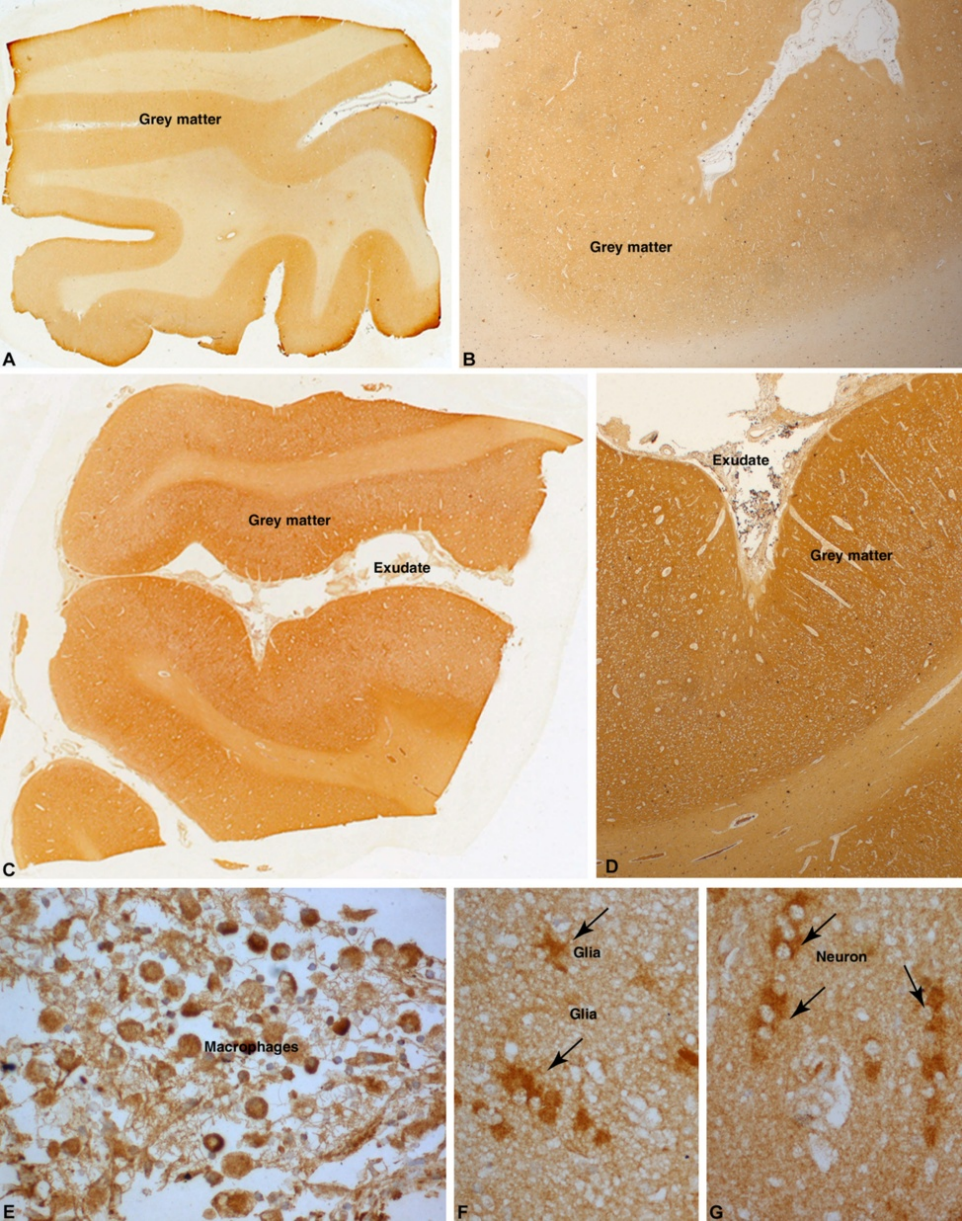
**Immunohistochemical labeling of NFASC protein overexpressed in TBM.** Normal controls (**A**, **B**): Light labeling of the cortical ribbon (**A**) in the frontal cortex with synaptic pattern (**B**). TBM(**C**-**G**): Whole mount preparation (**C**) highlight the darker labeling of the cortical ribbon and the inflammatory exudate in the subarachnoid space. The macrophages (**E**) entrapped in the inflammatory exudate are strongly labeled. Glial cells (**F**, arrows) and linearly arranged neuronal cytoplasm (**G**, arrows) are strongly labeled in the grey matter. [**A**, **C**: x10, B, **D**: xObj.5, **E**-**G**: xObj.20].

Neurofascin (NFASC) is an adhesion molecule, which plays an important role in cell communication and signal transduction. Overexpression of this protein was validated in all 15 TBM cases. NFASC in control cases revealed positive labeling of neuropil in cortex (Figure 5A, B) with only occasional neurons showing cytoplasmic labeling in the lower cortical layers. No labeling of astrocytes or oligodendroglia was noted in control cases. In cases of tuberculous meningitis, intense labeling was seen in the neuropil of grey matter in synaptic fashion reflecting upregulated expression of protein (Figure 5C, D). Several small granule neurons expressed cytoplasmic positivity in the upper cortical layers close to the surface exudates (Figure 5G). In addition, several reactive hypertrophic subpial astrocytes beneath the surface exudates expressed NFASC protein in cytoplasm extending into its branching processes (Figure 5F), in addition to macrophages in the inflammatory exudates in the subarachnoid space (Figure 5E).

**Figure 6:**
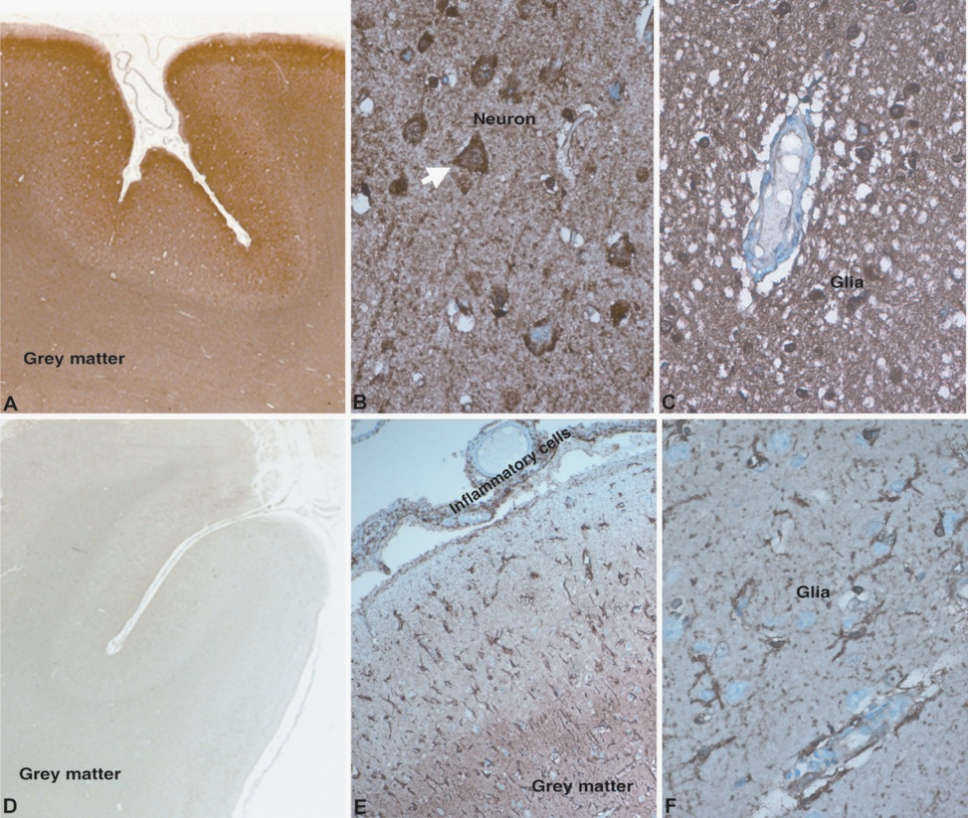
**Immunohistochemical labeling of FTL protein downregulation in TBM.** Normal controls (**A**-**C**): Intense labeling of grey and white matter (**A**). In the grey matter (**B**), the neurons (arrow) and dendritic processes are intensely labeled (**B**) while in the white matter (**C**), neuropil and oligodendroglial cells are stained intensely. TBM (**D**-**F**): In the grey and white matter of the frontal cortex, showed very light labeling in contrast to control. In the subpial zones and deep grey matter beneath the exudate and the inflammatory cells in the subarachnoid space are stained (**E**) while the neuropil and neurons are less intense. Microglial cells in white matter and perivascular zones show labeling (**F**). [**A**, **D**: whole mountsx10, **B**, **C**: xObj.20, **E**: xObj.x5, **F**: xObj.20].

Ferritin light chain (FTL) is an intracellular iron storage protein and downregulation of this protein was validated by IHC in 15 TBM cases (Figure 6). Expression of this protein was observed in control cases within the grey and white matter (Figure 6A), with grey matter showing higher intensity of expression compared to white matter. In the grey matter, neurons revealed intense labelling of the cytoplasm while dendritic processes in neuropil also revealed strong labelling (Figure 6B). In the white matter, oligodendroglial cells expressed the protein in its cytoplasm in addition to neuropil showing positivity (Figure 6C). In cases of tuberculous meningitis, marked down regulation of protein expression was seen in contrast to the normal cortex (Figure 6D). Interestingly, while neuronal expression was markedly downregulated, labelling was seen in microglial cells ensheathing neurons, as also in subpial and perivascular astrocytes seen below the inflammatory exudates in the subarachnoid spaces (Figure 6E). In the white matter, neuropil and perivascular microglia revealed protein expression in the branching ramified processes of activated miciroglia (Figure 6F).

### Public availability of proteomic data

We have submitted the peptide data to Human Proteinpedia (http://www.humanproteinpedia.org) [57] and raw data files to Tranche data repository (https://proteomecommons.org/tranche/) for easy access to the research community. The raw data is freely available in Tranche using the following hash:

xlm5a5zp5YBqpxdJpHYWlybPCdF9Bya2AH6AZRQ4dppr593JwMsptPIJCyVnJdbpOA+YHk3eNNQnNHdDQjeIH9c7QGsAAAAAAAFsYQ==

## Conclusions

The combination of mass spectrometry and quantitative proteomics is being increasingly utilized for discovering potential biomarkers. In the current study, using an iTRAQ-based quantitative proteomic approach, we were able to identify several known and novel molecules that are differentially expressed in TBM. We validated the expression levels of three novel biomarker candidates - AMPH, NFASC and FTL - by IHC on brain tissue sections from TBM cases as well as controls. These promising candidate markers warrant further evaluation in CSF from TBM patients to determine their clinical utility.

## Abbreviation

CSF: Cerebrospinal fluid; TBM: Tuberculous meningitis; iTRAQ: Isobaric tag for relative and absolute quantitation; QTOF: Quadrupole Time-of-Flight; IHC: Immunohistochemistry; TCEP: Tris (2-carboxyethyl) phosphine; MMTS: Methyl-methanethiosulfonate; TEAB: Triethylammonium bicarbonate buffer; ACN: Acetonitrile; Poly-HRP: Polymer conjugate of horseradish peroxidase; DAB-3: 3-Diaminobenzidine; HPLC: High performance liquid chromatography; SCX: Strong cation exchange; FDR: False discovery rate.

## Competing interests

The authors have declared no conflict of interest.

## Authors’ contributions

RC, TSKP, SKS and AP conceived the study, GSSK, YLR, PS, RC, TSKP, SKS and AP designed the experiments; GSSK, AKV, AM, SR, HCH, NAS, HP and RS, carried out the experiments; GSSK, SR, PK, RS, SRG, KW and AP analyzed the data; GSSK, SKS, and AP wrote the manuscript. All authors read and approved the final manuscript.

## Supplementary Material

Additional file 1 **Table S1.**List of TBM and control samples used in the present study. Click here for file

Additional file 2 **Table S2.**A complete list of Proteins identified in TBM. Click here for file

Additional file 3 **Table S3.**A complete list of peptides identified in TBM. Click here for file

## References

[B1] World Health Organization, Geneva, Switzerland The International Journal of Tuberculosis and Lung Disease 2006 10 1091 1097 17044200

[B2] Fanning A Tuberculosis: 6. Extrapulmonary disease CMAJ 1999 160 1597 1603 10374005 PMC1230370

[B3] Garg RK Tuberculosis of the central nervous system Postgrad Med J 1999 75 881 133 40 10448488 10.1136/pgmj.75.881.133 PMC1741157

[B4] Hosoglu S Geyik MF Balik I Predictors of outcome in patients with tuberculous meningitis Int J Tuberc Lung Dis 2002 6 1 64 70 11931403

[B5] Thwaites GE Nguyen DB Nguyen HD Dexamethasone for the treatment of tuberculous meningitis in adolescents and adults N Engl J Med 2004 351 17 1741 51 10.1056/NEJMoa040573 15496623

[B6] Berenguer J Moreno S Laguna F Tuberculous meningitis in patients infected with the human immunodeficiency virus N Engl J Med 1992 326 10 668 72 10.1056/NEJM199203053261004 1346547

[B7] Azuaje C Fernandez Hidalgo N Almirante B Tuberculous meningitis: a comparative study in relation to concurrent human immunodeficiency virus infection Enferm Infecc Microbiol Clin 2006 24 4 245 250 10.1016/S0213-005X(06)73770-3 16725084

[B8] Thwaites G Chau TT Mai NT Drobniewski F McAdam K Farrar J Tuberculous meningitis J Neurol Neurosurg Psychiatry 2000 68 3 289 99 10.1136/jnnp.68.3.289 10675209 PMC1736815

[B9] Dinnes J Deeks J Kunst H A systematic review of rapid diagnostic tests for the detection of tuberculosis infection Health Technol Assess 2007 11 3 1 196 17266837 10.3310/hta11030

[B10] Eintracht S Silber E Sonnenberg P Koornhof HJ Saffer D Analysis of adenosine deaminase isoenzyme-2 (ADA(2)) in cerebrospinal fluid in the diagnosis of tuberculosis meningitis J Neurol Neurosurg Psychiatry 2000 69 1 137 8 10.1136/jnnp.69.1.137 10864628 PMC1736999

[B11] Donald PR Malan C van der Walt A Schoeman JF The simultaneous determination of cerebrospinal fluid and plasma adenosine deaminase activity as a diagnostic aid in tuberculous meningitis S Afr Med J 1986 69 8 505 7 3961648

[B12] Mishra OP Loiwal V Ali Z Nath G Chandra L Cerebrospinal fluid adenosine deaminase activity for the diagnosis of tuberculous meningitis in children J Trop Pediatr 1996 42 3 129 32 10.1093/tropej/42.3.129 8699576

[B13] Kashyap RS Kainthla RP Mudaliar AV Purohit HJ Taori GM Daginawala HF Cerebrospinal fluid adenosine deaminase activity: a complimentary tool in the early diagnosis of tuberculous meningitis Cerebrospinal Fluid Res 2006 3 5 10.1186/1743-8454-3-5 16571142 PMC1448186

[B14] Nishida Y Komachi H Mizusawa H A case of Listeria meningitis associated with increased adenosine deaminase in cerebrospinal fluid Diagn Microbiol Infect Dis 2007 57 4 435 7 10.1016/j.diagmicrobio.2006.09.005 17141462

[B15] Mudaliar AV Kashyap RS Purohit HJ Taori GM Daginawala HF Detection of 65 kD heat shock protein in cerebrospinal fluid of tuberculous meningitis patients BMC Neurol 2006 6 34 10.1186/1471-2377-6-34 16978411 PMC1578580

[B16] Donald PR Malan C Cerebrospinal fluid lactate and lactate dehydrogenase levels as diagnostic aids in tuberculous meningitis S Afr Med J 1985 67 1 19 20 3966179

[B17] Molavi A LeFrock JL Tuberculous meningitis Med Clin North Am 1985 69 2 315 31 3990437 10.1016/s0025-7125(16)31045-8

[B18] Wilder-Smith A Wilder-Smith E Tuberculous meningitis and corticosteroids: a review Neurol J Southeast Asia 1998 3 57 60

[B19] Lorber J The results of treatment of 549 cases of tuberculous meningitis Am Rev Tuberc 1954 69 1 13 25 13114621 10.1164/art.1954.69.1.13

[B20] Verdon R Chevret S Laissy JP Wolff M Tuberculous meningitis in adults: review of 48 cases Clin Infect Dis 1996 22 6 982 8 10.1093/clinids/22.6.982 8783697

[B21] Chaerkady R Pandey A Applications of proteomics to lab diagnosis Annu Rev Pathol 2008 3 485 98 10.1146/annurev.pathmechdis.3.121806.151419 18039142

[B22] Han X Aslanian A Yates JR 3rd Mass spectrometry for proteomics Curr Opin Chem Biol 2008 12 5 483 90 10.1016/j.cbpa.2008.07.024 18718552 PMC2642903

[B23] Ackermann BL Hale JE Duffin KL The role of mass spectrometry in biomarker discovery and measurement Curr Drug Metab 2006 7 5 525 39 10.2174/138920006777697918 16787160

[B24] Chaerkady R Pandey A Quantitative proteomics for identification of cancer biomarkers Proteomics Clin Appl 2007 1 9 1080 9 10.1002/prca.200700284 21136759

[B25] Shiio Y Aebersold R Quantitative proteome analysis using isotope-coded affinity tags and mass spectrometry Nat Protoc 2006 1 1 139 45 10.1038/nprot.2006.22 17406225

[B26] Chaerkady R Harsha HC Nalli A A quantitative proteomic approach for identification of potential biomarkers in hepatocellular carcinoma J Proteome Res 2008 7 10 4289 98 10.1021/pr800197z 18715028 PMC3769105

[B27] Choe L D'Ascenzo M Relkin NR 8-plex quantitation of changes in cerebrospinal fluid protein expression in subjects undergoing intravenous immunoglobulin treatment for Alzheimer's disease Proteomics 2007 7 20 3651 60 10.1002/pmic.200700316 17880003 PMC3594777

[B28] Martin B Brenneman R Becker KG Gucek M Cole RN Maudsley S iTRAQ analysis of complex proteome alterations in 3xTgAD Alzheimer's mice: understanding the interface between physiology and disease PLoS One 2008 3 7 e2750 10.1371/journal.pone.0002750 18648646 PMC2453232

[B29] Pawar H Kashyap MK Sahasrabuddhe NA Quantitative tissue proteomics of esophageal squamous cell carcinoma for novel biomarker discovery Cancer Biol Ther 2011 12 6 510 22 10.4161/cbt.12.6.16833 21743296 PMC3218592

[B30] Prasad TSK Keerthikumar S Comparative proteomic analysis of Candida albicans and Candida glabrata Clinical Proteomics 2010 6 163 173 10.1007/s12014-010-9057-9

[B31] Ross PL Huang YN Marchese JN Multiplexed protein quantitation in Saccharomyces cerevisiae using amine-reactive isobaric tagging reagents Mol Cell Proteomics 2004 3 12 1154 69 10.1074/mcp.M400129-MCP200 15385600

[B32] Venugopal A Chaerkady R Pandey A Application of mass spectrometry-based proteomics for biomarker discovery in neurological disorders Ann Indian Acad Neurol 2009 12 1 3 11 20151002 10.4103/0972-2327.48845 PMC2811975

[B33] Yang W Woltjer RL Sokal I Quantitative proteomics identifies surfactant-resistant alpha-synuclein in cerebral cortex of Parkinsonism-dementia complex of Guam but not Alzheimer's disease or progressive supranuclear palsy Am J Pathol 2007 171 3 993 1002 10.2353/ajpath.2007.070015 17675576 PMC1959487

[B34] Zieske LR A perspective on the use of iTRAQ reagent technology for protein complex and profiling studies J Exp Bot 2006 57 7 1501 8 10.1093/jxb/erj168 16574745

[B35] Goel R Muthusamy B Pandey A Prasad TS Human protein reference database and human proteinpedia as discovery resources for molecular biotechnology Mol Biotechnol 2011 48 1 87 95 10.1007/s12033-010-9336-8 20927658

[B36] Garg A Barnes PF Porgador A Vimentin expressed on Mycobacterium tuberculosis-infected human monocytes is involved in binding to the NKp46 receptor J Immunol 2006 177 9 6192 8 17056548 10.4049/jimmunol.177.9.6192

[B37] Thuong NT Dunstan SJ Chau TT Identification of tuberculosis susceptibility genes with human macrophage gene expression profiles PLoS Pathog 2008 4 12 e1000229 10.1371/journal.ppat.1000229 19057661 PMC2585058

[B38] Beisiegel M Mollenkopf HJ Hahnke K Combination of host susceptibility and Mycobacterium tuberculosis virulence define gene expression profile in the host Eur J Immunol 2009 39 12 3369 84 10.1002/eji.200939615 19795415

[B39] Kumar GSS Venugopal AK Selvan LDN Marimuthu A Keerthikumar S Pathare S Dikshit JB Tata P Hariharan R Prasad TSK Harsha HC Ramachandra YL Mahadevan A Chaerkady R Shankar SK Pandey A Gene expression profiling of tuberculous meningitis J Proteomics Bioinformatics 2011 4 5 98 105

[B40] Wu CJ Chen Z Ullrich A Greene MI O'Rourke DM Inhibition of EGFR-mediated phosphoinositide-3-OH kinase (PI3-K) signaling and glioblastoma phenotype by signal-regulatory proteins (SIRPs) Oncogene 2000 19 35 3999 4010 10.1038/sj.onc.1203748 10962556

[B41] Stofega MR Argetsinger LS Wang H Ullrich A Carter-Su C Negative regulation of growth hormone receptor/JAK2 signaling by signal regulatory protein alpha J Biol Chem 2000 275 36 28222 9 10842184 10.1074/jbc.M004238200

[B42] Kharitonenkov A Chen Z Sures I Wang H Schilling J Ullrich A A family of proteins that inhibit signalling through tyrosine kinase receptors Nature 1997 386 6621 181 6 10.1038/386181a0 9062191

[B43] Neznanov N Neznanova L Kondratov RV O'Rourke DM Ullrich A Gudkov AV The ability of protein tyrosine phosphatase SHP-1 to suppress NFkappaB can be inhibited by dominant negative mutant of SIRPalpha DNA Cell Biol 2004 23 3 175 82 10.1089/104454904322964779 15068587

[B44] Neznanov N Neznanova L Kondratov RV Dominant negative form of signal-regulatory protein-alpha (SIRPalpha/SHPS-1) inhibits tumor necrosis factor-mediated apoptosis by activation of NF-kappa B J Biol Chem 2003 278 6 3809 15 10.1074/jbc.M210698200 12446684

[B45] Hayano T Kikuchi M Cloning and sequencing of the cDNA encoding human P5 Gene 1995 164 2 377 8 10.1016/0378-1119(95)00474-K 7590364

[B46] Berwin B Floor E Martin TF CAPS (mammalian UNC-31) protein localizes to membranes involved in dense-core vesicle exocytosis Neuron 1998 21 1 137 45 10.1016/S0896-6273(00)80521-8 9697858

[B47] Renden R Berwin B Davis W Drosophila CAPS is an essential gene that regulates dense-core vesicle release and synaptic vesicle fusion Neuron 2001 31 3 421 37 10.1016/S0896-6273(01)00382-8 11516399

[B48] Wigge P Kohler K Vallis Y Amphiphysin heterodimers: potential role in clathrin-mediated endocytosis Mol Biol Cell 1997 8 10 2003 15 9348539 10.1091/mbc.8.10.2003 PMC25662

[B49] Vozzolo L Loh B Gane PJ Gyrase B inhibitor impairs HIV-1 replication by targeting Hsp90 and the capsid protein J Biol Chem 2010 285 50 39314 28 10.1074/jbc.M110.155275 20937817 PMC2998086

[B50] Zonta B Tait S Melrose S Glial and neuronal isoforms of Neurofascin have distinct roles in the assembly of nodes of Ranvier in the central nervous system J Cell Biol 2008 181 7 1169 77 10.1083/jcb.200712154 18573915 PMC2442198

[B51] Zonta B Desmazieres A Rinaldi A A critical role for Neurofascin in regulating action potential initiation through maintenance of the axon initial segment Neuron 2011 69 5 945 56 10.1016/j.neuron.2011.02.021 21382554 PMC3057015

[B52] Koticha D Babiarz J Kane-Goldsmith N Jacob J Raju K Grumet M Cell adhesion and neurite outgrowth are promoted by neurofascin NF155 and inhibited by NF186 Mol Cell Neurosci 2005 30 1 137 48 10.1016/j.mcn.2005.06.007 16061393

[B53] Friedman A Arosio P Finazzi D Koziorowski D Galazka-Friedman J Ferritin as an important player in neurodegeneration Parkinsonism Relat Disord 2011 6 423 430 21550835 10.1016/j.parkreldis.2011.03.016

[B54] Maresca V Flori E Cardinali G Ferritin light chain down-modulation generates depigmentation in human metastatic melanoma cells by influencing tyrosinase maturation J Cell Physiol 2006 206 3 843 8 10.1002/jcp.20543 16252260

[B55] Roeser H Jacobs A Worwood M Jacobs A, Worwood M Iron metabolism in biochemistry and medicine. Volume 2 1980 605 640

[B56] Kwak EL Larochelle DA Beaumont C Torti SV Torti FM J Biol Chem 1995 270 15285 15293 10.1074/jbc.270.25.15285 7797515

[B57] Kandasamy K Keerthikumar S Goel R Human Proteinpedia: a unified discovery resource for proteomics research Nucleic Acids Res 2009 37 Database issue D773 781 18948298 10.1093/nar/gkn701 PMC2686511

