# Supplementary material for: Quantitative proteomics for identifying biomarkers for tuberculous meningitis
Source: Clin Proteomics. 2012 Nov 30;9(1):12. doi: 10.1186/1559-0275-9-12 (PMC3572431; doi:10.1186/1559-0275-9-12)
Supplement: Additional file 1 — Table S1.List of TBM and control samples used in the present study. [file 1559-0275-9-12-S1.doc]

| **S.No** | **Sample ID** | **Age/Sex** | **Post mortem details** | **Diagnosis** | **Tissue Source** |
| --- | --- | --- | --- | --- | --- |
| 1 | **Control_1** | 23/M | 6h.20min | Normal | Frontal cortex |
| 2 | **Control_2** | 24/M | 22h | Normal | Frontal cortex |
| 3 | **Control_3** | 35/M | 15h.30min | Normal | Frontal cortex |
| 4 | **Control_4** | 40/M | 7h.15min | Normal | Frontal cortex |
| 5 | **Control_5** | 25/F | 8 hrs | Normal | Frontal cortex |
| 6 | **Control_6** | 25/M | 1.30 hrs | Normal | Frontal cortex |
| 7 | Control_7 | ……… | ……… | Normal | Frontal cortex |
| 8 | Control_8 | ……… | ……… | Normal | Frontal cortex |
| 9 | Control_9 | 13/M | 15 hrs | Normal | Frontal cortex |
| 10 | Control_10 | 5 ½/F | ……… | Normal | Frontal cortex |
| 11 | Control_11 | 38/M | 10 hrs | Normal | Frontal cortex |
| 12 | Control_12 | 55/M | 7 hrs | Normal | Frontal cortex |
| 13 | Control_13 | 40/F | 7 hrs 15 min | Normal | Frontal cortex |
| 14 | Control_14 | 9/M | ……… | Normal | Frontal cortex |
| 15 | Control_15 | 28/M | 19 hrs 30 min | Normal | Frontal cortex |
| 16 | Control_16 | 27/F | 4 hrs | Normal | Frontal cortex |
| 17 | **TBM_1** | 60/M | 14 hrs | TBM | Frontal cortex |
| 18 | **TBM_2** | 25/F | 9 hrs 15 min | TBM | Frontal cortex |
| 19 | **TBM_3** | 44/M | 14 hrs | TBM | Frontal cortex |
| 20 | **TBM_4** | 20/M | 1 hr 15 min | TBM | Frontal cortex |
| 21 | **TBM_5** | 16/M | 3 hrs 30 min | TBM | Frontal cortex |
| 22 | **TBM_6** | 25/M | ……… | TBM | Frontal cortex |
| 23 | TBM_7 | 30/M | 13 hrs | TBM | Frontal cortex |
| 24 | TBM_8 | 30/M | 4 hrs | TBM | Frontal cortex |
| 25 | TBM_9 | 2/F | 1 hr | TBM | Frontal cortex |
| 26 | TBM_10 | 30/M | 18 hrs | TBM | Frontal cortex |
| 27 | TBM_11 | 28/M | 5 hrs 15 min | TBM | Frontal cortex |
| 28 | TBM_12 | 13/M | ……… | TBM | Frontal cortex |
| 29 | TBM_13 | 20/F | ……… | TBM | Frontal cortex |
| 30 | TBM_14 | 48/M | 18 hrs | TBM | Frontal cortex |
| 31 | TBM_15 | 35/F | 11 hrs | TBM | Frontal cortex |
| **Note:** Serial no 1-16 belongs to control cases. 1-6 cases (bold) were used for iTRAQ experiments and 7-16 cases were used for IHC experiments. Serial no 17-31 belongs to the infectious samples. 17-22 cases (bold) were used for iTRAQ as well as for IHC experiments. Cases 23-31 were used for IHC experiments. The cases selected as controls are negative for neuroinfection by serology, gross examination and histological examination. The diagnosis of TBM was established by histological demonstration of granulomatous meningitis and encephalitis, demonstration of acid fast bacilli organism in the brain smear by Ziehl–Neelsen stain, mycobacterial antibody and/or mycobacterial immune complexes/or mycobacterial culture. | | | | | |

**Supplementary Table 1: List of TBM and control samples used in the present study**
